# *Ophelimus bipolaris* sp. n. (Hymenoptera, Eulophidae), a New Invasive *Eucalyptus* Pest and Its Host Plants in China †

**DOI:** 10.3390/insects12090778

**Published:** 2021-08-30

**Authors:** Hua-Yan Chen, Jie-Min Yao, Shao-Bin Huang, Hong Pang

**Affiliations:** 1 State Key Laboratory of Biocontrol, School of Life Sciences/School of Ecology, Sun Yat-sen University, Guangzhou 510275, China; chenhuayan@mail.sysu.edu.cn (H.-Y.C.); Lsshpang@mail.sysu.edu.cn (H.P.); 2 Guangdong Eco-Engineering Polytechnic, Guangzhou 510520, China; h3602@126.com

**Keywords:** Chalcidoidea, DNA barcoding, exotic pest, gall wasp, new species

## Abstract

**Simple Summary:**

*Eucalyptus* species have become one of the most commonly planted trees worldwide, including China. However, the productivity of *Eucalyptus* plantations has been threatened by the recent increase of invasive insect pests. Gall inducers of the genus *Ophelimus* (Eulophidae) are among the most important invasive species in *Eucalyptus* plantations. Based on the combined analysis of biological, morphological and molecular evidence, we here describe a new invasive species, *Ophelimus bipolaris* sp. n., from China. This wasp induces galls only on the leaf blade surface of four *Eucalyptus* species. It can complete a life cycle on *E. urophylla* in approximately 2 months under local climatic conditions in Guangzhou, China.

**Abstract:**

*Eucalyptus* species have become one of the most commonly planted trees worldwide, including China, due to their fast growth and various commercial applications. However, the productivity of *Eucalyptus* plantations has been threatened by exotic invasive insect pests in recent years. Among these pests, gall inducers of the genus *Ophelimus* of the Eulophidae family are among the most important invasive species in *Eucalyptus* plantations. We report here for the first time the presence of a new invasive *Eucalyptus* gall wasp, *Ophelimus bipolaris* sp. n., in Guangzhou, China, which also represents the first species of the genus reported from China. The identity of the new species was confirmed by an integrative approach combing biological, morphological and molecular evidence. The new species is described and illustrated. This wasp induces galls only on the leaf blade surface of four *Eucalyptus* species: *E. grandis*, *E. grandis* × *E. urophylla*, *E. tereticornis* and *E. urophylla.* Our preliminary observation showed that *O. bipolaris* could complete a life cycle on *E. urophylla* in approximately 2 months under local climatic conditions (23.5–30 °C). Considering the severe damage it may cause to *Eucalyptus* production, further investigations of its biology and control are urgently needed in China.

## 1. Introduction

Most species of the genus *Eucalyptus* (Myrtaceae) are native to Australia, but have been commonly planted worldwide due to their fast growth and various commercial applications [1]. Eucalypts were first introduced to China sometime before 1894 [2], and the expansion of plantations has dramatically increased in the country since the 1980s [3]. By 2017, eucalypt plantations had been established in all provinces of China south of the Yangtze River, and these plantations amounted to 5.4 M ha [4].

**Figure 1:**
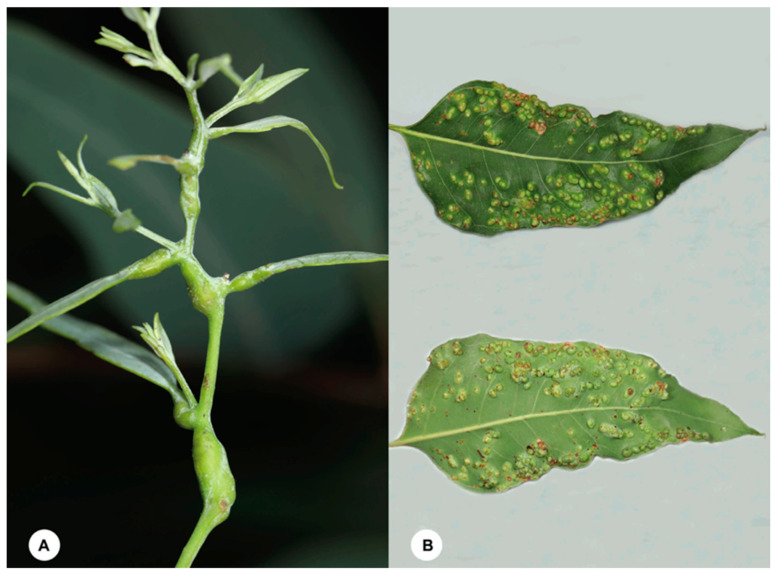
(**A**) Galls of *Leptocybe invasa* Fisher & La Salle on *Eucalyptus exserta* F. Muell. (**B**) Galls of *Ophelimus bipolaris* sp. n. on *Eucalyptus urophylla* S. T. Blake. (All photographed in Guangzhou, China).

**Figure 2:**
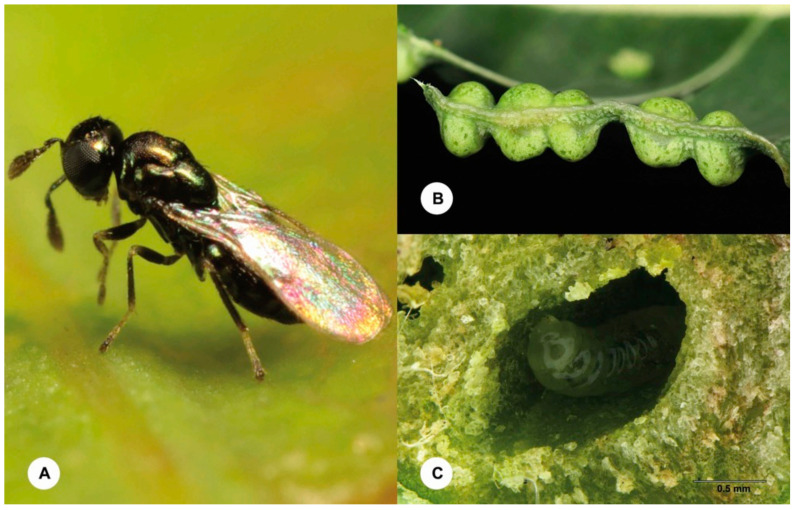
(**A**) A female of *Ophelimus bipolaris* sp. n. on the young leaf of *Eucalyptus urophylla* S. T. Blake. (**B**) Mature galls on the leaf edge of *E. urophylla*, lateral view (**C**) A mature gall on the leaf of *E. urophylla* cut open to show the larva of *Ophelimus bipolaris* sp. n.

In China, there are about 300 species of phytophagous insects associated with eucalypts [5], including the invasive gall-inducer, *Leptocybe invasa* Fisher & La Salle (Hymenoptera, Eulophidae), which was first reported from China in 2007 [6]. Although at least one molecular study [7] has suggested that *L. invasa* in fact comprises two cryptic species, with the female-biased population from China being genetically different from the first-described thelytokous population from the Mediterranean region, no formal taxonomic act has been proposed for the species complex. Nevertheless, *Leptocybe invasa* is currently the only gall-forming pest of eucalypts recorded in China, and it forms galls (Figure 1A) on the stems, petioles and midribs of leaves of a few *Eucalyptus* species in the sections *Exertaria*, *Latoanulata* and *Maidenaria* [8]. In April 2021, we found a new form of protruding galls (Figure 1B) on the leaves (never on mid-ribs or branches) of *Eucalyptus urophylla* S. T. Blake on the campus of Guangdong Eco-Engineering Polytechnic, Guangzhou, China. At the site of the infected trees, we also observed that wasps belong to the family Eulophidae (Hymenoptera) were apparently laying eggs on the young leaves (Figure 2A). On examination of the specimens collected from the leaves and reared from the galls, we found that these wasps are conspecific and belong to the genus *Ophelimus* Haliday.

The genus *Ophelimus* is native to Australia and currently contains 53 described species [9,10]. Available biological data indicate that species of the genus develop in galls on various species of *Eucalyptus* and are considered gall inducers [11,12]. Infestations of these gall inducers, especially those invasive species occur outside their native range, can lead to intense gall production on *Eucalyptus* trees and subsequently, severe defoliation, causing significant economic losses [13,14,15]. Originally described as *Rhicnopeltella eucalyptis* Gahan [16] based on female specimens reared from galls on *Eucalyptus globulus* Labill from New Zealand in 1922, *Ophelimus eucalypti* (Gahan) was considered the first invasive species reported outside its native Australian origin [13]. By 1987, this species had been recorded inducing galls on the midribs and branches of eucalypt species in the section *Maidenaria,* and no males of these populations had been observed, and therefore, populations of the species that infected the section *Transversaria* were later considered the ‘Maid’ biotype [13]. In 1987, another population identified as *O. eucalypti* in New Zealand was reared from eucalypt species in the section *Transversaria* and induced galls only on the leaf blade surface. This latter population was biparental and later was considered as the ‘Trans’ biotype [13]. Borowiec et al. [9] recently confirmed that *O. eucalypti* comprises two cryptic lineages (‘Maid and ‘Trans’) based on the host plant, reproduction mode and morphological and molecular (*28S*) differences. *Ophelimus eucalypti* was erroneously reported in Europe [9,14], but by 2019, both lineages of *O. eucalypti* were only listed in New Zealand. Recently, the first report of *O. eucalypti* outside of New Zealand was in Sumatra, Indonesia, where it had caused serious damage to *E. urophylla* and hybrids of the species with *Eucalyptus grandis* W. Hill, but the lineage has not been determined [15]. However, currently the most widely distributed invader is *Ophelimus maskelli* (Ashmead), which was first described as *Pteroptrix maskelli* from New Zealand by Ashmead in 1900 and was transferred to the genus *Ophelimus* by Bouček [11]. Outside its native range, *O. maskelli* has been reported from the Mediterranean Basin, Southeast Asia, South Africa and North America (see references in Borowiec et al. [9] and Dittrich-Schröder et al. [15]). *Ophelimus maskelli* is a thelytokous (only reproduce females) species and induces blister-like galls near the petiole on the leaf blade of *Eucalyptus* species. Uncontrolled populations of these wasps can cause severe leaf damage and almost complete defoliation of mature trees in some cases [14,17]. Fourteen host species have been recorded for *O. maskelli*, with *Eucalyptus camaldulensis* Dehnhardt and *Eucalyptus tereticornis* Smith being economically important and particularly suitable species [14]. Recently, two species, *Ophelimus mediterraneus* Borowiec & Burks and *Ophelimus migdanorum* Molina-Mercader were newly described from the Mediterranean Europe [9] and Chile [10], respectively. *Ophelimus mediterraneus* is also a thelytokous species and induces galls on the upper surface of the leaves on *Eucalyptus* species from the *Maidenaria* section, such as *Eucalyptus globulus* Labill and *Eucalyptus gunii* J. D. Hook [9]. While *O. migdanorum* is a biparental species and induces galls on stems, petioles, laminae and leaf venations of *E. globulus* and *Eucalyptus camaldulensis* Dehnh [10].

Considering the economic importance of *Ophelimus* species to the production of *Eucalyptus* trees, in this study, we aim to investigate the identity of the *Ophelimus* species we just found in Guangzhou, China, using an integrative taxonomic approach combining biological, morphological and molecular information.

## 2. Materials and Methods

### 2.1. Insect Sampling

The initial survey was conducted between April and July 2021 in a small *E. urophylla* plantation on the campus of Guangdong Eco-Engineering Polytechnic (GEEP), Guangzhou, China. To investigate the host range of the wasp, additional surveys were conducted between early June and late July at three other localities in Guangzhou (Table 1). Wasps on the leaves of *Eucalyptus* trees were collected and preserved in 95% ethanol. Mature leaves with large galls of each infected *Eucalyptus* species were collected, labeled and placed in small plastic bags at the laboratory of Sun Yat-sen University, Guangzhou, China. Emergences were checked daily, and all emerged adult wasps were collected in 95% ethanol to allow for further molecular and morphological study. Voucher specimens are deposited in the Museum of Biology at Sun Yat-sen University (SYSBM), Guangzhou, China. During the initial survey in April at the plantation of GEEP, young leaves of *E. urophylla* were observed being attacked by the wasps. Such leaves were recorded and left on the tree until they were covered with large mature galls and then were collected in plastic bags as described above.

### 2.2. Species Identification

Morphological terminology generally follows Gibson et al. [18]. The systematics and taxonomy of *Ophelimus* are poorly studied [14]. However, some characters, especially the number of setae on the submarginal vein of fore wings, are thought to be diagnostically valuable [14]. Recently, Borowiec et al. [9] provided a key for some *Ophelimus* species of agricultural interest. To supplement morphological identifications, two molecular markers, mitochondrial DNA (mtDNA) cytochrome c oxidase 1 (*COI*) and nuclear *28S* rRNA D1–2 (*28S*) were sequenced for molecular species delimitation. Genomic DNA was extracted from 13 adults and two larvae dissected from the gall using a nondestructive method as described in Taekul et al. [19]. Detailed information about the sequenced specimens used in this study is given in Table 2. Polymerase chain reaction (PCR) amplifications of the two DNA fragments were performed using Tks Gflex DNA Polymerase (Takara, Shiga, Japan) and conducted in a T100 Thermal Cycler (Bio-Rad). The primer pairs LCO1490/HCO2198 [20] and D2-3551F/D2-4057R [21] were used for *COI* and *28S*, respectively. Thermocycling conditions were: an initial denaturing step at 94 °C for 5 min, followed by 35 cycles of 94 °C for 30 s, 50 °C for 30 s, 72 °C for 30 s and an additional extension at 72 °C for 5 min. Amplicons were directly sequenced in both directions with forward and reverse primers on an Applied Biosysttems (ABI) 3730XL (Applied Biosystems, Foster City, CA, USA) by Guangzhou Tianyi Huiyuan Gene Technology Co., Ltd. (Guangzhou, China). Chromatograms were assembled with Geneious 11.0.3. All the amplified sequences were deposited into GenBank (Table 2).

All sequences were blasted in the BOLD (Barcode of Life Database, http://www.barcod-inglife.org/index.php/IDS_OpenIdEngine, only for *COI*) and GenBank. The sequences generated in this study along with representatives generated by Molina-Mercader et al. [10] and Borowiec et al. [9] were aligned using MAFFT v7.470 by the Q-INS-I strategy for *28S* and G-INS-I strategy for *COI* [22]. After removing the identical sequences, the alignments were then analyzed using RAxML as implemented in Geneious 11.0.3. Sequences of *Closterocerus chamaeleon* (Girault) (Hymenoptera: Eulophidae) were used as outgroups to root the trees as used by Borowiec et al. [9].

### 2.3. Photography

Images of live specimens and trees were taken with a Canon 5D Mark III (Tokyo, Japan) camera with a 100 mm macro lens. Images of mounted specimens were produced using a Nikon SMZ25 microscope (Melville, NY, USA) with a Nikon DS-Ri 2 (Melville, NY, USA) digital camera system. Images of the type specimen of *O. eucalypti* were provided by the National Museum of Natural History (NMNH), Smithsonian Institution, Washington, DC, USA. Scanning electron micrographs were produced using a Phenom Pro Desktop SEM and single montage images were generated from image stacks in the program Helicon. Images were post-processed with Adobe Photoshop CS6 Extended.

## 3. Results

Of the four sampled localities, six *Eucalyptus* species or hybrid species were surveyed and four of them were infected by *Ophelimus* wasps: *E. grandis*, *E. grandis* × *E. urophylla*, *E. tereticornis* and *E. urophylla* (Table 1). Galls were only found on the leaf blade surface of all the four infected *Eucalyptus* species. Mature galls (Figure 2B) are 2–3 mm in diameter and protrude 1–2 mm on each side of the leaf. Each gall contains a single larva (Figure 2C) but eggs tend to be laid close together on a leaf and develop patches of tightly packed galls. In severe cases, the entire leaf is totally covered with galls. Galls change from green to red, then to brown. A circular exit hole is left on the gall as the adult wasp emerges. In this study, a total of 1244 *Ophelimus* specimens were collected, and 97.2% were females. Detailed information about the collected specimens is given in Table S1.

During the survey, at the end of April at the GEEP plantation, young leaves of the upper shoots of five two-year old trees at the infested site showed no sign of galls, but by the end of May, almost each of those leaves was covered with numerous green or red galls, and a few adult wasps emerged in mid-June. According to the temperature records provided by the China Meteorological Data Service Center, the average temperatures of Guangzhou from April to July ranged from 23.5 °C to 30 °C (Figure S1). Although biological studies of this wasp species on the host plants are still on going, our preliminary observation showed that the duration of its life cycle is approximately 2 months, at least so on *E. urophylla* in Guangzhou.

**Figure 3:**
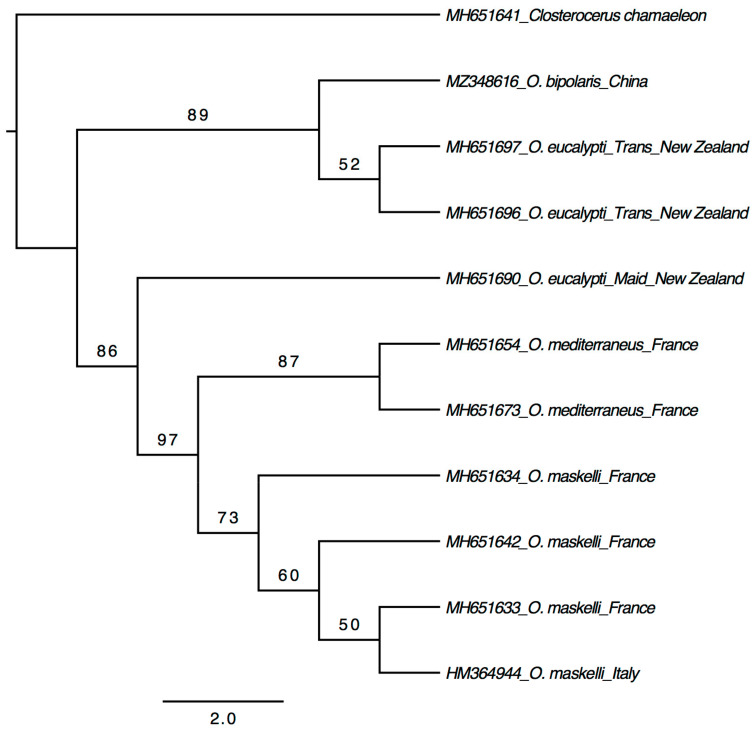
Maximum likelihood tree based on *28S*.

**Figure 4:**
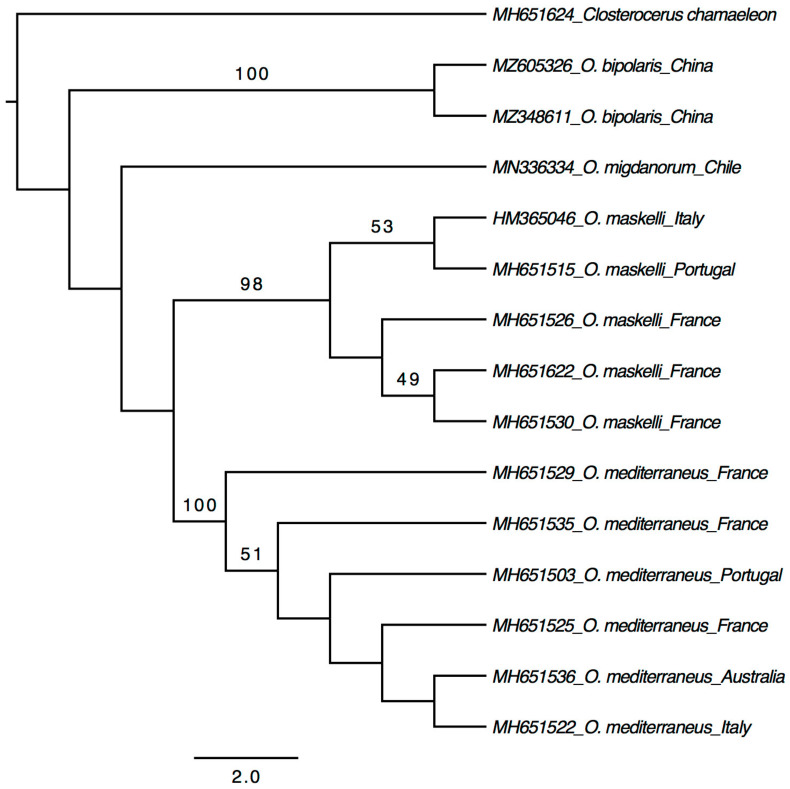
Maximum likelihood tree based on *COI*, only values >50 for bootstrap are labeled.

Both *28S* and *COI* genes were successfully sequenced from all the 15 specimens (13 adults + 2 larvae). All the sequences of *28S* (605 bp) were identical to each other and showed 99.3–99.5% identity to the *O. eucalypti* ‘Trans’ biotype and 98.1% to the *O. eucalypti* ‘Maid’ biotype in the GenBank database (Table S2). Phylogenetic analysis based on *28S* sequences generated from this study together with those used by Borowiec et al. [9] showed that the Chinese *Ophelimus* species is sister to the *O. eucalypti* ‘Trans’ biotype (Figure 3), which together form a clade clearly separated from other species. The 15 sequences of *COI* (660–678 bp) were also mostly identical, with only one sequence (MZ348610) differed by two nucleotides. These *COI* sequences do not show a high match with sequences in both the BOLD and GenBank databases. The closest match is *Ophelimus migdanorum* Molina-Mercader, with 92.28% identical pairs of bases (Table S3). When analyzed with the *COI* sequences of *O. maskelli* (Ashmead), *O. mediterraneus* and *O. migdanorum*, the two unique *COI* sequences of the Chinese *Ophelimus* species formed a clade sister to *O. migdanorum* but with a low support (Figure 4). While there is no *COI* sequence of either biotype of *O. eucalypti* in the BOLD and GenBank databases, the *28S* sequences well suggest that the Chinese *Ophelimus* species might be conspecific to the *O. eucalypti* ‘Trans’ biotype or a closely related species. Considering that the two biotypes of *O. eucalypti* are most likely two distinct species, as confirmed by Borowiec et al. [9] using molecular, morphological (number of submarginal vein setae) and ecological (host range) data, the *Ophelimus* species we found in Guangzhou should represent a distinct species different from *O. eucalypti sensu* Gahan.

Further examination indicates that the wasps we collected in Guangzhou are morphologically identical. By comparing the holotype of *O. eucalypti* (based on images provided by NMNH, Figure S2) and the original description of the species provided by Gahan [16], as well as running the key compiled by Borowiec et al. [9], we conclude that the *Ophelimus* species we found belong to an undescribed species and we here describe it as new to science below.

### Ophelimus bipolaris Chen & Yao, sp. n.

Material examined: Holotype, female, CHINA: Guangdong, Guangzhou, campus of Guangdong Eco-Engineering Polytechnic, 23°11′58″ N 113°22′35″ E, reared from galls on the leaf of *Eucalyptus urophylla* S. T. Blake, 24.iv.2021, Huayan Chen, HC739 (deposited in SYSBM) (GenBank accession numbers: *COI*, MZ348610; *28S*, MZ348616). Paratypes: (116 females, 12 males) CHINA: 50 females, 10 males, same data as holotype (SYSBM); 20 females, 2 males, Guangdong, Guangzhou, Xiaoguwei Island, 23°4′0″ N 113°22′41″ E, reared from galls on the leaf of *E. urophylla*, 11.vii.2021, Huayan Chen (SYSBM); 20 females, Guangdong, Guangzhou, Xiaoguwei Island, 23°4′0″ N 113°22′41″ E, reared from galls on the leaf of *E. grandis* × *urophylla*, 11.vii.2021, Huayan Chen (SYSBM); 4 females, Guangdong, Guangzhou, South China Botanical Garden, 23°10′52″ N 113°21′28″ E, reared from galls on the leaf of *E. tereticornis*, 13.vii.2021, Huayan Chen (SYSBM); 15 females, Guangdong, Guangzhou, Huolushan Forest Park, 23°10′39″ N 113°22′56″ E, reared from galls on the leaf of *E. grandis*, 13.vii.2021, Huayan Chen (SYSBM); 7 females, Guangdong, Guangzhou, Huolushan Forest Park, 23°10′39″ N 113°22′56″ E, reared from galls on the leaf of *E. urophylla*, 13.vii.2021, Huayan Chen (SYSBM).

Etymology: The name *bipolaris* refers to the gall induced by this species that protrudes from both sides of the leaves of the host plants.

Diagnosis. Submarginal vein of fore wing with 3–5 dorsal setae. Body mainly reticulate. Mesoscutal midlobe with 5 pairs of long setae. Propodeum medially longer than metascutellum. Marginal vein about 1.8 × length of stigmal vein. Postmarginal vein distinctly shorter than stigmal vein. The new species is similar to other well-known *Ophelimus* invasive species. The differences between the new species and other four *Ophelimus* species of agriculture interest are summarized in Table 3.

**Figure 5:**
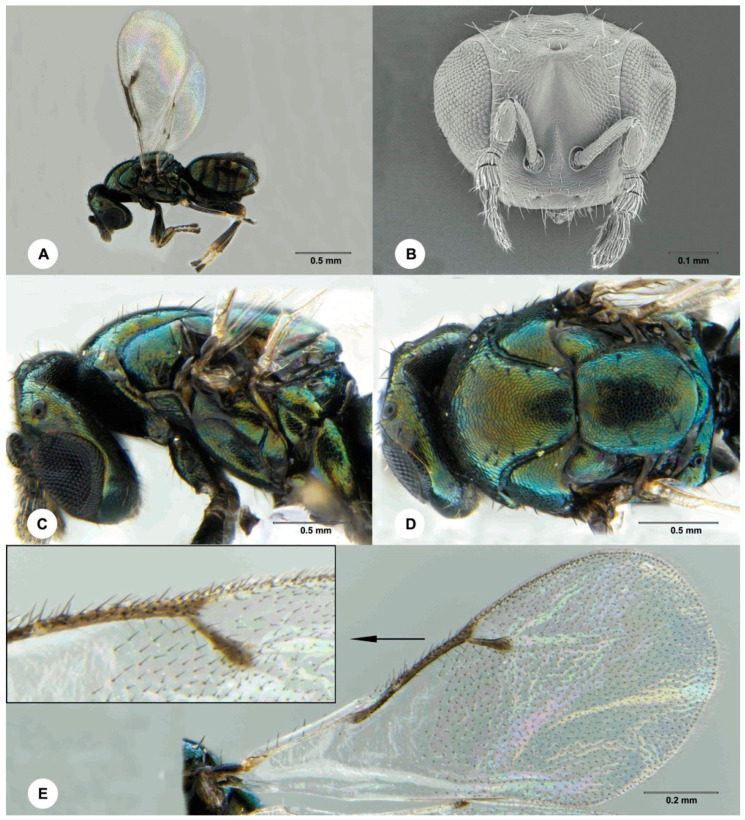
*Ophelimus bipolaris* sp. n., female (**A**) Habitus, lateral view (**B**) Head, anterior view (**C**) Head and mesosoma, lateral view (**D**) Head and mesosoma, dorsal view (**E**) Fore wing.

Description: Female (Figure 5). Body length 1.1–1.8 mm.

Colour: Head and body brown with variable metallic green and orange luster, metasoma darker dorsally. Antenna brown. Coxae brown with metallic green luster, first three tarsomeres pale brown, remainders of legs dark brown to brown. Wings hyaline, with veins grayish black.

Head: Reticulate, except scrobal depression and clypeus smooth. Vertex, gena, lateral frons and ventral half of face with sparse long setae. Ocelli in a low triangle, widely separated, the posterior ocelli separated from the eye margin by about the diameter of an ocellus. Eye with short setae that are visible at high magnification. Malar sulcus shallow but visible. Clypeus small, lateral margin hardly distinguishable. Anterior tentorial pit present. Mandible bidentate, ventral tooth much larger than dorsal tooth.

Antenna: Pedicel slight shorter then funicle. First four flagellomeres anellifrom, the last also transverse but much larger and bearing multiporous plate sensilla. Club longer than the other flagellmeres combined, ovate, with three distinct clavomeres, the apical one bearing a long terminal seta.

Mesosoma: Entirely reticulate. Mesoscutum slightly longer than mesoscutellum. Mesoscutal midlobe with 5 pairs of long setae, anterior 2 pairs of setae relatively shorter. Notaulus deep and complete, sharply curved outward anteriorly. Mesoscutellum slightly longer than broad, with 2 pairs of long setae. Mesoscutellar rim not carinate, but slightly overhanging metascutellum. Axillular groove present. Metascutellum short, not overhanging propodeum. Propodeum medially longer than metascutellum, posterior margin excavated medially, lateral propodeal area with 3–5 setae.

Wings: Fore wing about as long as body, about twice as long as broad. Submarginal vein with 3–5 dorsal setae. Marginal vein about 1.8 × length of stigmal vein. Postmarginal vein distinctly shorter than stigmal vein, tapering gradually from the base until lost in the margin of wing.

Legs: Coxae reticulate. Femora and tibia imbricate. Tibial spur formula 1-1-1. Mesotibial spur about as long as the first two mid tarsomeres combined. Hind femur slightly thickened. Hind tibia densely setose.

Metasoma: Subspherical, about as long as mesosoma or slightly shorter, apex not pointed. First tergum the longest, second to sixth terga subequal, each about half length of the first tergum. Ovipositor short, not exserted. Hypopygium reaching about 0.3 × length of metasoma. All terga reticulate.

**Figure 6:**
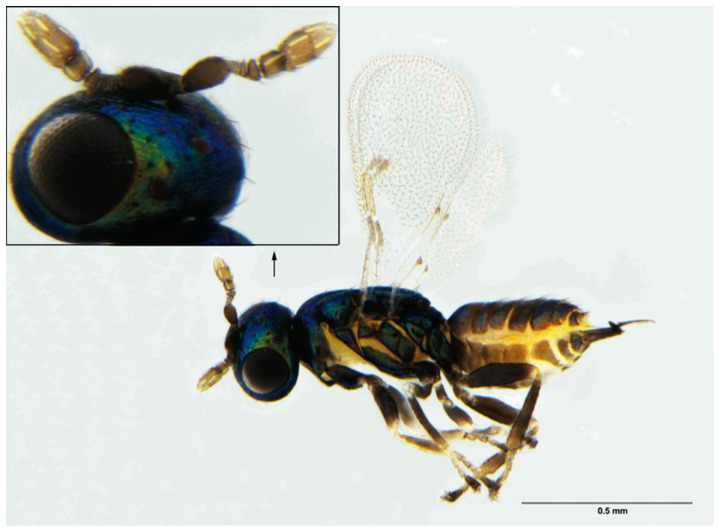
*Ophelimus bipolaris* sp. n., male, habitus with head in the excerpt, lateral view.

**Figure 7:**
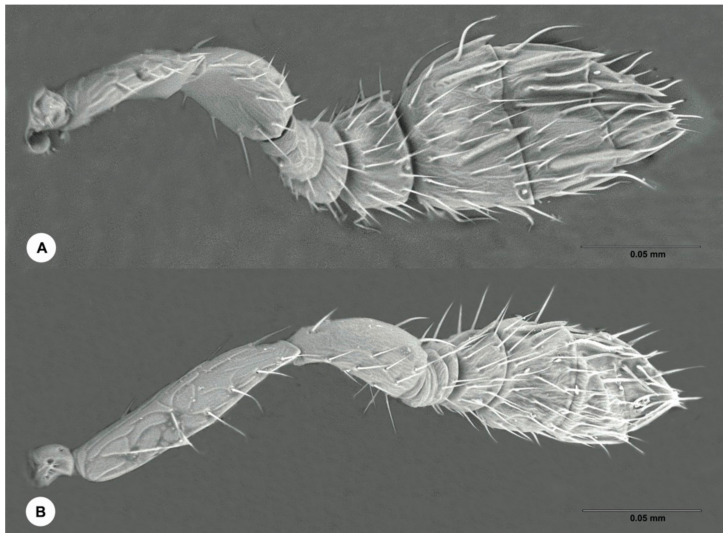
*Ophelimus bipolaris* sp. n. Antenna (**A**) Female (**B**) Male.

Male (Figure 6): Body length 1.0–1.2 mm. Similar to female, except: submarginal vein of fore wing with 3–4 setae; antennal club relatively slender and covered with fewer multiporous plate sensilla (Figure 7); genitalia extruded.

Hosts: *E. grandis*, *E. grandis* × *E. urophylla*, *E. tereticornis* and *E. urophylla*.

Distribution: China (Guangdong).

## 4. Discussion

There are 53 described species of *Ophelimus* and the genus is poorly studied and needs a thorough taxonomic revision [9,14]. Therefore, synonymies are likely to be detected in future. It is possible that *O. bipolaris* was described previously under another name. However, considering the fact that the descriptions of most of the old species are always poor and the types are in very bad conditions [9], the task of attempting to attain the holotype of each species to rule out that possible species’ identity would severely delay or even prohibit the execution of this study. The combined analyses of biological, molecular and morphological data (Table 3) presented here should permit the unequivocal identification of *O. bipolaris* sp. n.

*Ophelimus bipolaris* induces protruding galls on the leaves of *E. grandis*, *E. grandis* × urophylla, *E. tereticornis* and *E. urophylla*. The galls induced by *O. bipolaris* are most similar to those induced by the *O. eucalypti* ‘Trans’ biotype (Table 3). However, gall morphology is different between females and males of the *O. eucalypti* ‘Trans’ biotype, with females inducing circular, protruding galls and males inducing pit galls, while galls induced by *O. bipolaris* show no differences between both sexes. The host range of *O. bipolaris* is also similar to the *O. eucalypti* ‘Trans’ biotype, which has been reported to attack Eucalyptus species in the section *Transversaria* and *E. urophylla* [13,15]. However, one of the host plants of *O. bipolaris*, *E. tereticornis*, is in the *Dumaria* section of *Eucalyptus*.

The reproduction modes are different among *Ophelimus* species, but all seem to be female-biased. The *O. eucalypti* ‘Maid’ biotype [24], *O. maskelli* [14] and *O. mediterraneus* [9] have been reported as thelytokous that reproduce females only. According to Withers et al. [13], the *O. eucalypti* ‘Trans’ biotype is biparental, but the sex ratio was not clearly stated in their study, although Dittrich-Schröder et al. [15] erroneously claimed that the lineage was male-biased when citing Withers et al.’s study. *Ophelimus migdnorum* is also biparental, and 58.9% are females [10]. Our study indicates that *O. bipolaris* is female-biased, with about 97.2% of the collected specimens being females. Female-biased sex ratio occurs frequently in Chalcidoidea, and it has been associated with infection by symbiotic bacteria able to manipulate the reproduction of their host [25]. Sex ratio variations might reflect the infection by different bacterial endosymbionts, resulting in different reproduction modes of the hosts and therefore different species lineages. For example, molecular analyses suggested that *L. invasa* is in fact a complex of two cryptic species that are infected by two closely related strains of *Rickettsia* [7]. Therefore, screening for the infection of endosymbionts among *Ophelimus* species, especially the two biotypes of *O. eucalypti* and *O. bipolaris*, is a possible direction in clarifying the identities of these species.

Both the *28S* and *COI* sequences showed unambiguous differentiation between *O. bipolaris* and four other species (Figure 3 and Figure 4), although the *28S* sequences of *O. bipolaris* and the *O. eucalypti* ‘Trans’ biotype are 99.3–99.5% identical, and one might suspect that *O. bipolaris* is conspecific with the *O. eucalypti* ‘Trans’ biotype. However, the *28S* is conserved and often invariant between closely related species in Eulophidae [26,27]. The low distance of the *28S* sequences among *Ophelimus* species is consistent with what already observed in Eulophidae [9,28]. While the divergence of *COI* sequences is high between *O. bipolaris* and other studied *Ophelimus* species, most of the *COI* sequences are identical among the specimens collected from all the four studied localities. Even the two sequences have only two different nucleotides. This reduced genetic *COI* variation could be due to founder effects (the reduction in genetic variation that results when a small subset of a large population is used to establish a new colony) [29] or endosymbiont infection (endosymbionts can act as reproductive manipulators and are considered responsible for the low mitochondrial genetic diversity in infected populations) [30,31]. Further studies are required to investigate the cause of this low mitochondrial genetic diversity.

The result of the morphological analysis (Table 3) was also consistent with the molecular results, indicating *O. bipolaris* is a distinct species. The number of setae on the submarginal vein of the fore wing was first thought be an important diagnostic character for *Ophelimus* species [14,32], at least *O. maskelli* was thought to be the only species with one single submarginal vein seta, but subsequent studies showed that this character was, however, not discriminant among *Ophelimus* species [9,10]. According to Molina-Mercader et al. [10], the number of submarginal vein setae is in accordance with the body size of the specimen. *Ophelimus bipolaris* has 3–5 submarginal vein setae, and we indeed found that smaller specimens tend to have fewer setae (Table S4). Body size was used in the key compiled by Borowiec et al. [9], but apparently this character is also not discriminant among *Ophelimus* species. Besides, body size is easily affected by temperature and the host plant species [33]. Nevertheless, *O. eucalypti* is the largest species recorded, and *O. bipolaris* is relatively smaller. Body color seems to be useful in separating *O. bipolaris* (head and mesosoma are metallic green) from *O. eucalypti* (head and mesosoma mainly black and only faintly tinged with metallic green or purplish). The following characters might be of diagnostic value: (1) the postmarginal vein is much shorter than stigmal vein; (2) the mesoscutal midlobe with 5 pairs of long setae; (3) the propodeum is distinctly longer than metascutellum medially.

Our preliminary observation showed that *O. bipolaris* on *E. urophylla* only took approximately 2 months to complete a life cycle in Guangzhou, under local climatic conditions (temperature: 23.5–30 °C). Obviously, its life cycle might be affected by temperature and host plant species, as has been found in *O. maskelli* [14]. Further studies regarding the host range and life cycle of *O. bipolaris* in China are required.

The origin of *O. bipolaris* is unknown, but undoubtedly it originates from Australia or Indonesia, since it exclusively attacks *Eucalyptus* species. Of its four known host plant species or hybrids, *E. tereticornis* and *E. tereticornis* are native to Australia, while *E. urophylla* is native to Indonesia. Therefore, *O. bipolaris* is an invasive species in China. As mentioned above, the low mitochondrial genetic diversity may be due to founder effects, and it may suggest that this is a recent invasion in China. Considering the severe damage and economic loss of *Eucalyptus* have been caused by the invasive species of *Ophelimus* outside their native ranges [9,10,13,14,34], eradication or control strategies against *O. bipolaris* is urgently needed in China.

## 5. Conclusions

Based on the result of analyzing the biological, morphological and molecular evidence, we have formally described a new invasive species of the *Eucalyptus* gall wasp, *Ophelimus bipolaris* Chen & Yao, which represents the first species of the genus present in China. This wasp induces protruding galls only on the leaf blade of *Eucalytpus*. Its host plants at least include *E. grandis*, *E. grandis* × *E.* urophylla, *E. tereticornis* and *E. urophylla* in China. Our preliminary observation showed that *O. bipolaris* can complete a life cycle on *E. urophylla* in approximately 2 months under local climatic conditions. Further studies on the life cycle, host range, geographical distribution, economical damage and management of this wasp are urgently needed in China and possible distributed countries.

## Figures and Tables

**Table 1:** Details of surveyed localities and *Eucalyptus* species.

Locality	Coordinates	*Eucalyptus* Species
Guangdong Eco-Engineering Polytechnic (GEEP)	23°11′58″ N 113°22′35″ E	*E. urophylla* *
Xiaoguwei Island (XI)	23°4′0″ N 113°22′41″ E	*E. urophylla* *, *E. grandis* × *E. urophylla* *, *E. citriodora*, *E. exserta*
South China Botanical Garden (SCBG)	23°10′52″ N 113°21′28″ E	*E. tereticornis* *, *E. citriodora*, *E. exserta*
Huolushan Forest Park (HFP)	23°10′39″ N 113°22′56″ E	*E. urophylla* *, *E. grandis* *, *E. exserta*

* Denotes the species is infected by *Ophelimus* species.

**Table 2:** Details of specimens sequenced and accession numbers.

Code	Locality	Host Plant	Stage	Sex	Submarginal Vein Setae	GenBank Accession Number	
						*COI*	*28S*
HC739	GEEP	*E. urophylla*	adult	female	4	MZ348610	MZ348616
HC740	GEEP	*E. urophylla*	larva	NA	NA	MZ348611	MZ348617
HC745	GEEP	*E. urophylla*	adult	male	3	MZ348612	MZ348618
HC746	GEEP	*E. urophylla*	adult	male	4	MZ348613	MZ348619
HC747	GEEP	*E. urophylla*	adult	female	5	MZ348614	MZ348620
HC785	XI	*E. grandis* × *E. urophylla*	adult	female	4	MZ605326	MZ605354
HC786	XI	*E. grandis* × *E. urophylla*	larva	NA	NA	MZ605327	MZ605355
HC787	XI	*E. urophylla*	adult	female	5	MZ605328	MZ605356
HC788	XI	*E. urophylla*	adult	female	4	MZ605329	MZ605357
HC789	HFP	*E. grandis*	adult	female	4	MZ605330	MZ605358
HC790	HFP	*E. grandis*	adult	female	3	MZ605331	MZ605359
HC791	HFP	*E. urophylla*	adult	female	5	MZ605332	MZ605360
HC792	HFP	*E. urophylla*	adult	female	4	MZ605333	MZ605361
HC793	XI	*E. urophylla*	adult	male	3	MZ605334	MZ605362
HC794	SCBG	*E. tereticornis*	adult	female	4	MZ605335	MZ605363

**Table 3:** Comparison of *Ophelimus bipolaris* with other known *Ophelimus* species.

	*O. bipolaris*	*O. eucalypti* ‘Maid.’	*O. eucalypti* ‘Trans.’	*O. maskelli*	*O. mediterraneus*	*O. migdnorum*
Head color	Brown with metallic green	Mainly black, frons faintly tinged with metallic green	Mainly black, frons faintly tinged with metallic green	Brown with metallic green	Brown with metallic green	Brown with metallic green
Mesosoma color	Brown with metallic green	Mainly black, dorsal mesosoma faintly tinged with purplish	Mainly black, dorsal mesosoma faintly tinged with purplish	Brown with metallic green	Brown with metallic green	Brown with metallic green
Body length	Female: 1.1–1.8 mm; male: 1.0–1.2 mm	Female: 2.0–2.5 mm	Female: 2.0–2.5 mm; male: ?	Female: 0.8–1.1 mm	Female: 0.8–1.0 mm	Female: 0.7–1.4 mm; male: 0.7–1.4 mm
No. of setae on submarginal vein	3–5	2–4	≥5	1	2–4	1–3
Marginal vein/Stigmal vein	About 1.8 ×	About 2 ×	About 2 × ?	About 0.7×	About 0.7×	0.7–0.8×
Postmarginal vein/Stigmal vein	<0.5×	>1×	>1 × ?	>2×	>1×	>3×
No. of setae on mesoscutal midlobe	5 pairs	6 pairs	6 pairs?	2 pairs	2 pairs	Unknown
Propodeum vs. Metascutellum	Distinctly longer	Subequal	Subequal?	Subequal	Subequal	Unknown
Hosts	*E. grandis*, *E. grandis* × *urophylla*, *E. tereticornis* and *E. urophylla*	*Eucalyptu* from section *Maidenaria*	*Eucalyptu* from section *Transversaria*	*Eucalyptu* from 3 sections: *Exsertaria*, *Latoangulata* and *Maidenaria*	*Eucalyptu* from section *Maidenaria*	*E. globulus*, *E. camaldulensis*
Galls	Only on leaf blade, round and smooth, green then to reddish galls visible on both sides of the leaves	On leaf midribs, leaf blade and shoot axes, round and smooth, green then to reddish galls	Only on leaf blade, females induce circular, protruding galls, males induce pit galls	Only on leaf blade, round and smooth, green then to reddish galls visible on both sides of the leaves	Only on leaf blade, ellipsoidal, conical shaped, brown coloured with rough and racked surface on just the upper side of the leaves	On leaf blade, midrib, secondary rib, petiole, and twigs, amorphous

Note: “?” denotes uncertain because the *O. eucalypti* ‘Trans.’ biotype has been reported indistinguishable from *O. eucalypti* ‘Maid.’ biotype but never been clearly described. Summarized data of body measurements and best ratios *of O. bipolaris* see Table S4. Data of *O. eucalypti*, *O. maskelli*, *O. mediterraneus* and *O. migdnorum* are from published literatures [9,10,13,14,16,23].

## Data Availability

The data of the research were deposited in the Museum of Biology at Sun Yat-sen University (SYSBM), Guangzhou, China.

## Supplementary Materials

The following are available online at https://www.mdpi.com/article/10.3390/insects12090778/s1. Figure S1. Average temperatures of Guangzhou from April to July, 2021. (Data from China Meteorological Data Service Center). Figure S2. *Rhicnopeltella eucalyptis* Gahan, holotype, female A Habitus, dorsal view B Habitus, lateral view C Head and mesosoma, dorsal view D Head and mesosoma, lateral view E Head, anterior view F Wings. (Images are used with permission from NMNH). Table S1. Details of the sampling, host plant and number of wasps collected. Table S2. Interspecific pairwise distance of *Ophelimus* species based on *28S* sequences (%). Table S3. Interspecific pairwise distance of *Ophelimus* species based on *COI* sequences (%); Table S4. Summarized data of body measurements (in mm) and best ratios of *O. bipolaris*.
