# Supplementary material for: Ophelimus bipolaris sp. n. (Hymenoptera, Eulophidae), a New Invasive Eucalyptus Pest and Its Host Plants in China
Source: Insects. 2021 Aug 30;12(9):778. doi: 10.3390/insects12090778 (PMC8465356; doi:10.3390/insects12090778)
Supplement: Supplementary file 1 [file insects-12-00778-s001.zip › insects-1278981-supplementary.pdf]

Supplementary Material:

Table S1. Details of the sampling, host plant and number of wasps collected.

Locality	Coordinates	Host plant	Collecting method	No. of wasps Female	Male
Guangdong Eco-Engineering Polytechnic (GEEP)	23°11'58" N 113°22'35" E	*E. urophylla*	reared from galls	1014	33
		E. urophylla	direct catch	15	0
Xiaoguwei Island (XI)	23°4'0" N 113°22'41" E	*E. urophylla*	reared from galls	107	2
		E. grandis × *E. urophylla*	reared from galls	47	0
South China Botanical Garden (SCBG)	23°10'52" N 113°21'28" E	*E. tereticornis*	reared from galls	4	0
Huolushan Forest Park (HFP)	23°10'39" N 113°22'56" E	*E. grandis*	reared from galls	15	0
		E. urophylla	reared from galls	7	0

Table S2. Interspecific pairwise distance of *Ophelimus* species based on *28S* sequences (%).

Species	1	2	3	4	5
1 *O. bipolaris*		1.9	0.5–0.7	2.5	2.5
2 *O. eucalypti* "Maid"			1.3–1.5	1	1
3 *O. eucalypti* "Trans"				2–2.2	2–2.2
4 *O. maskelli*					0.3–1.7
5 *O. mediterraneus*					

Table S3. Interspecific pairwise distance of *Ophelimus* species based on *COI* sequences (%).

Species	1	2	3	4
1 *O. bipolaris*		16.1–18.4	9.3–18.1	7.7
2 *O. maskelli*			7.5–28.6	9.7–26.9
3 *O. mediterraneus*				8.4–8.9
4 *O. migdanorum*				

Table S4. Summarized data of body measurements (in mm) and best ratios of *O. bipolaris*.

Character	female												male							
	3 setae (n=8)				4 setae (n=24)				5 setae (n=9)				3 setae (n=14)				4 setae (n=13)			
	min	max	mean	SD	min	max	mean	SD	min	max	mean	SD	min	max	mean	SD	min	max	mean	SD
BL	1.1	1.6	1.29	0.178	1.2	1.8	1.52	0.184	1.32	1	1.58	0.157	1	1.2	1.1	0.06	1.01	1.2	1.11	0.07
MvL	0.194	0.214	0.206	0.008	0.198	0.22	0.212	0.005	0.212	0.219	0.214	0.002	0.194	0.21	0.204	0.007	0.195	0.21	0.203	0.006
StvL	0.11	0.12	0.113	0.003	0.101	0.128	0.116	0.007	0.113	0.124	0.119	0.003	0.11	0.115	0.112	0.002	0.11	0.115	0.112	0.002
PmvL	0.037	0.049	0.041	0.004	0.04	0.051	0.046	0.003	0.042	0.051	0.048	0.003	0.036	0.042	0.039	0.002	0.036	0.042	0.039	0.002
MetL	0.022	0.027	0.024	0.002	0.023	0.029	0.026	0.002	0.022	0.029	0.026	0.002	0.023	0.026	0024	0.001	0.023	0.027	0.024	0.001
PpL	0.046	0.052	0.05	0.002	0.049	0.058	0.052	0.003	0.049	0.057	0.052	0.003	0.048	0.052	0.05	0.001	0.047	0.052	0.05	0.001
Best Ratios																				
MvL/StvL	1.764	1.875	1.82	0.046	1.719	1.96	1.828	0.071	1.766	1.876	1.8	0.033	1.764	1.875	1.816	0.037	1.772	1.842	1.814	0.024
PmvL/StvL	0.336	0.408	0.36	0.022	0.368	0.413	0.395	0.012	0.372	0.413	0.4	0.016	0.327	0.368	0.35	0.014	0.327	0.368	0.346	0.013
PpL/MetL	1.96	2.174	2.091	0.074	1.857	2.167	2.026	0.104	1.857	2.227	2.022	0.112	1.962	2.174	2.088	0.064	1.926	2.217	2.056	0.084

Note: BL=body length; MvL=marginal vein length; StvL=stigmal vein length; PmvL=postmarginal vein length; MetL=metascutellum length; PpL=propodeum length.

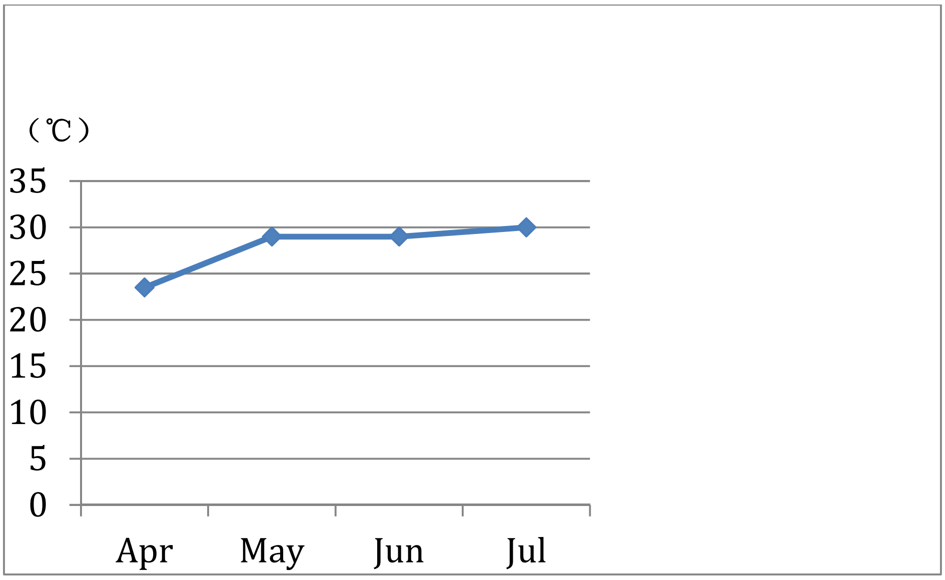

Figure S1. Average temperatures of Guangzhou from April to July, 2021. (Data from China Meteorological Data Service Center.)

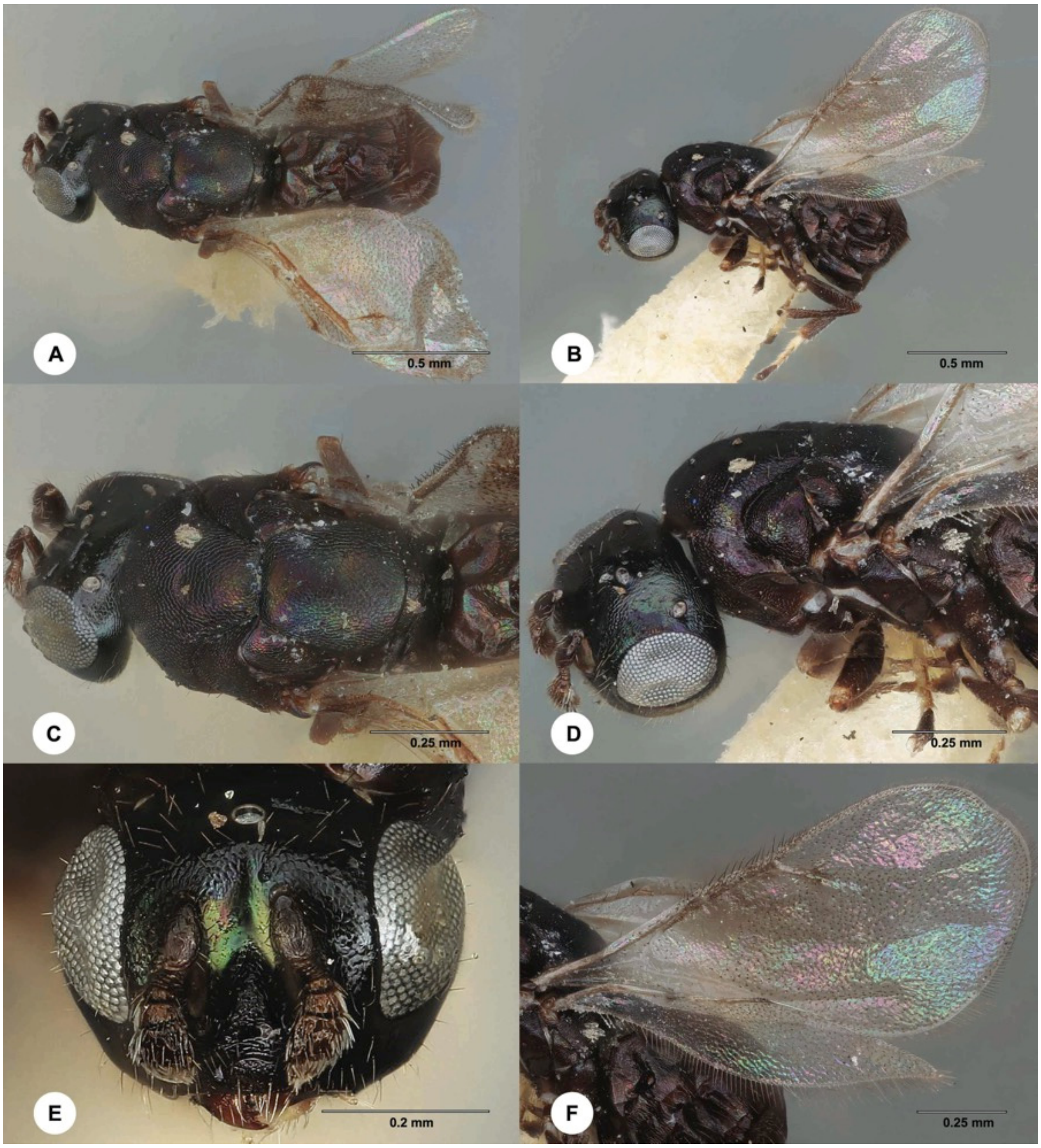

Figure S2. *Rhicnopeltella eucalyptis* Gahan, holotype, female **A** Habitus, dorsal view **B** Habitus, lateral view **C** Head and mesosoma, dorsal view **D** Head and mesosoma, lateral view **E** Head, anterior view **F** Wings. (Images are used with permission from NMNH.)
